# The Effectiveness of Platelet-Rich Plasma in the Treatment of Sciatic Nerve Injury: A Single-Blind Randomized Comparative Trial

**DOI:** 10.1155/np/7540054

**Published:** 2025-11-18

**Authors:** Congmin Yang, Changji Wang, Chaoyang Wang, Guan Yang, Wei Wu

**Affiliations:** Department of Pain Medicine, Chinese People's Liberation Army Western Theater General Hospital, Chengdu, China

**Keywords:** peripheral nerve injury, platelet-rich plasma, rehabilitation training, sciatic nerve injury

## Abstract

**Purpose:**

To evaluate the efficacy and safety of platelet-rich plasma (PRP) in treating sciatic nerve injury (SNI).

**Methods:**

A prospective, randomized, single-blind, comparative trial was conducted. Thirty patients with SNI were randomized into two groups of 15, namely, the PRP and control groups. In the PRP group, patients were injected with 5 doses of 3 mL PRP combined with 12 weeks of rehabilitation training using ultrasound guidance, while the control group received 12 weeks of rehabilitation training. Motor function recovery rating table (MFRRT) and sensory function recovery rating table (SFRRT) were used as primary outcomes. The secondary outcomes included the cross-sectional area (CSA) of the sciatic nerve under ultrasound guidance and electrophysiological assessment. Evaluations were performed at baseline and 1–, 3-, and 6-month postinjection.

**Results:**

After treatment, there were significant differences in the motor function recovery rating, motor conduction velocity, sensory conduction velocity, and CSA of the sciatic nerve at 1, 3, and 6 months in the PRP group (*p* < 0.05). There were significant differences in the motor conduction velocity of the sciatic nerve at 6 months in the control group (*p* < 0.05).

**Conclusions:**

PRP may be partially effective in the early repair of incomplete sciatic nerve injuries, and its efficacy could be maintained.

## 1. Introduction

Sciatic nerve injury (SNI) is a transient or lifelong sensory and motor nerve dysfunction caused by traction, compression, or ischemia. This is a peripheral nerve injury. Studies have shown that the number of sciatic nerve injuries in China is as high as hundreds of thousands every year, mostly among young and middle-aged men, and the trend is rising significantly, However, only 10%–25% of the patients have fully recovered from the injury, which seriously affects the patients' daily life and their ability to participate in society, and creates a huge economic burden for the family and society [1–3]. Therefore, research on repair and functional recovery after peripheral nerve injury is urgent.

Currently, the treatment of peripheral nerves mainly includes surgical treatment, rehabilitation treatment, drug treatment, and biological treatment [4–9]. Surgical treatment [4] was able to restore the nerve connection; the nerve repair could only rely on the natural growth of the nerve after the surgery, while the surgery brings local injuries, scar proliferation, graft rejection, and other undesirable complications to the growth of the nerve. Rehabilitation treatments included exercise, acupuncture, and electrical stimulation to slow down muscle atrophy, but did not directly promote nerve regeneration [5]. Pharmacological treatments included glucocorticoid therapy, nutritional nerve therapy, and murine nerve growth factor, etc., which were used to improve the local microenvironment through drugs to provide corresponding support for nerve growth, but this indirect effect was difficult to quickly improve the nerve function [6, 7]. Biological therapy included the local application of various types of stem cells, nerve growth factor, etc. [8, 9]. Such treatments were mostly in the stage of experimental research, and their efficacy was unstable, safety needed to be confirmed, and cell therapy faced many problems, such as cell source, cell survival ability in the local aggregation, tumorigenicity, and so on.

Platelet-rich plasma (PRP) is a concentrate of autologous platelets that releases a variety of growth factors to promote tissue regeneration. This method does not require special equipment or training, and is cost-effective and valuable in regenerative medicine [10]. There is evidence of PRP's potential to promote nerve regeneration: (1) neuroprotection and prevention of neuronal apoptosis; (2) stimulation of vascular regeneration; (3) promotion of axonal regeneration; (4) regulation of the inflammatory response in the microenvironment; (5) alleviation of nerve collateral muscle atrophy; and (6) improvement of human nervous system parameters [11]. However, clinical studies on PRP have focused on the peripheral nerves of the upper limbs, and their results showed good recovery of nerve function, with fewer reports on the sciatic nerve after injury. Therefore, we hypothesized that PRP may help repair SNI and improve sensory and motor functions of the lower limbs. We compared the safety and efficacy of PRP with those of traditional rehabilitation therapy in patients with SNI to provide more evidence for its clinical application.

## 2. Materials and Methods

### 2.1. Study Design

Altogether, 30 male patients diagnosed with unilateral SNI were eligible for inclusion in the study between March 2022 and December 2023. Thirty patients were separately randomized into either the PRP or the control group. The patients in the PRP group were treated with five injections of 5 mL PRP and rehabilitation, and those in the control group were treated with rehabilitation. All participants were followed up for 6 months. All patients were instructed not to receive other SNI treatments for 2 weeks before and during the study period, including physical therapy and neurotrophic factor injection, except for acetaminophen (500 mg, up to 4 g/d) as a rescue medication. An investigator regularly monitored patients for the use of other types of therapies during the study period.

### 2.2. Diagnostic Criteria

The diagnostic criteria for SNI in neurology [12] were as follows: (1) history of local injury, (2) hypokinesia, muscle atrophy, lower limb deformities, and impaired motor function, (3) dysfunction of sensory innervation, (4) reduced skin temperature, skin atrophy, little or no sweating, and decreased or even absent tendon reflexes in the innervated area, and (5) diagnosis confirmed with the aid of electromyography and ultrasound of the musculoskeletal system.

### 2.3. Inclusion and Exclusion Criteria

The inclusion criteria for our study were meeting the diagnostic criteria. The injury was located in the middle of the posterior thigh, on the affected side. Male aged 18–60 years. The course was 3 weeks after an incomplete SNI. The patient had not received any other treatment before the trial. The oral medication was simply a methylcobalamin tablet for neurotropism. Patients without other diseases were included. We excluded patients with a diagnosis or history of the following: abnormal sciatic nerve function in both lower limbs before the trauma; severe cardiopulmonary, hematological, skin, infectious, oncological, and psychiatric disorders.

## 3. PRP Preparation

The elbow vein blood (100 mL) was placed into a centrifuge tube with preheparinized treatment, centrifuged at 2500 r/min for 15 min, and the middle layer was used to obtain PRP. This PRP was processed again in the same way, combined with the PRP obtained from the two centrifugations, and placed in a refrigerator at −80°C for storage [13].

### 3.1. Perineural Injection With PRP

**Figure 1:**
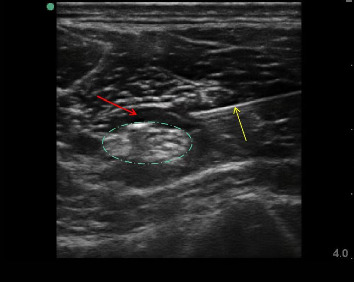
Ultrasound-guided imaging of platelet-rich plasma injections in the sciatic nerve. The high-echo image indicated by the yellow arrow shows the injection needle, the echo-less image indicated by the red arrow shows platelet-rich plasma, and the high-echo image within the blue oval line indicates the sciatic nerve.

An independent physician with 5 years of experience performed the ultrasound-guided perineural injections. The patient was in prone position, used the American Sonosite portable high-frequency ultrasonic instrument, select high-frequency line array probe (6–12 Hz) or low-frequency convex array probe if necessary placed the ultrasound probe at the injured nerve, the ultrasound image can be clearly shown in the pike shaped and high glossy sciatic nerve, mark the skin, used in-plane technique after local anesthesia, the puncture needle (Henan Shuguang Huizhikang Bio-technology Co., Ltd, single-use sterile syringe with needle 5 mL–0.5 × 36,) reached the nerve periphery, drawed back no blood, inject 5 mL PRP, ultrasound showed that the liquid wrapt around the nerve, and withdrawed the needle. Inject 1 time per week, continuous treatment for 5 times (Figure 1). Rehabilitation was the same as in the control group.

### 3.2. Rehabilitation Training

Depending on the condition, the patients were instructed to perform active and passive sensory training and homework training five times per week for 12 weeks.

### 3.3. Outcome Measurements

All outcome measurements were assessed by another single physician, who was blind to group assignments. Assessments were measured before treatment and at 1, 3, and 6 months after treatment.

## 4. Primary Outcome

### 4.1. Motor Function Recovery Rating Table (MFRRT) and Sensory Function Recovery Rating Table (SFRRT) From the British Medical Research Council (BMRC) After Peripheral Nerve Injury

All cases were assessed by the MFRRT and SFRRT developed by the BMRC [14]. MFRRT: M0, no muscle contraction; M1, contraction of proximal muscles is visible; M2, contraction of both proximal and distal muscles is visible; M3, all muscles are able to resist contraction; M4, all activities can be performed, including independent and coordinated movements; and M5, completely normal. Sensory recovery scale rating criteria: S0, no recovery of sensation; S1, recovery of deep skin sensation in the innervated area; S2, partial recovery of cutaneous pain and touch in the innervated area; S3, recovery of cutaneous pain and touch in the innervated area and disappearance of sensory hypersensitivity; S3+, recovery of two-point discrimination sensation after reaching the level of S3; S4, complete recovery. The BMRC classifies motor and sensory function recovery grade criteria into four levels: excellent (≥M4S3+), good (M3S3), moderate (M2S2), and poor (M0-1S0-1).

## 5. Secondary Outcomes

### 5.1. Cross-Sectional Area of the Sciatic Nerve (mm^2^)

**Figure 2:**
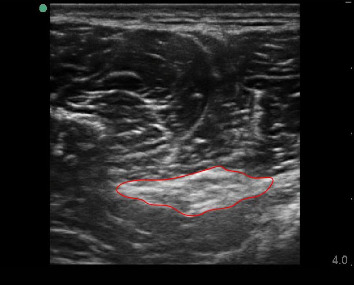
Ultrasound measurement of the cross-sectional area of the sciatic nerve. The high-echo imaging within the red irregular line indicates the cross-sectional area.

The patient was in a supine position, the operator sat in front of the patient, and the ultrasound equipment and probe were the same as above. The 6 Hz high-frequency line array probe was placed on the injured side, and the probe was perpendicular to the nerve course. At this time, the ultrasound image showed the transverse axis of the nerve (Figure 2). The image was swept from the distal to the proximal end to mark the site of the nerve injury. The cross-sectional area of the nerve was measured 3 times, and the average value was recorded.

### 5.2. Electrophysiological Study

The motor and sensory conduction velocities of the bilateral sciatic nerves were detected using electromyographic evoked potentials from Nikolai, USA [15]. The stimulation intensity was 20–99 mA, the frequency was 1 Hz, the scanning speed of the motor conduction assay was 2 ms/D, the sensitivity was 5 m V/D, the scanning speed of the sensory conduction assay was 2 ms/D, the sensitivity was 10 μV/D, and the filtering range was 20–10 kHz. The skin temperature was measured to maintain 32°C, the skin was treated with a scrub to reduce the impedance, the tibial nerve and common peroneal nerve were detected by motor conduction, and the sural nerve and superficial peroneal nerve were detected by sensory conduction. The nerve conduction velocity was generally considered abnormal if it was outside the normal range of mean values.

### 5.3. Safety Evaluation

Liver and kidney function, routine blood tests, electrocardiograms, and other indexes were included, and treatment-related adverse reactions were recorded.

### 5.4. Sample Size [16]

Paired *t*-test was used for power analysis by comparing the intergroup improvements in the BMRC table between preinjection and 1, 3, and 6 months post-injection. Based on this result, at least 15 cases per group were required to reach the necessary statistical power ([1-*β*] = 0.9; *α* = 0.05; effect size ranging from 0.5 to 0.9).

### 5.5. Statistical Analysis

The data were analyzed with the statistical software SPSS 23.0. Chi-square or Fisher's exact test was used to analyze continuous and categorical demographic data. Two-way ANOVA multiple comparisons were performed for the intragroup comparisons at follow-ups. The paired *t*-test was performed for the intergroup comparisons. *p*-Values <0.05 were considered statistically significant (two-tailed).

## 6. Results

30 patients completed the study (Table 1). Table 1 shows the baseline demographics and clinical characteristics of the patients in the two groups; there were no significant differences between the groups. Compared with the baseline, there were significant differences in the MFRRT of the common peroneal nerve at 3 and 6 months and the MFRRT and SFRRT of the tibial nerve at 6 months after treatment in the PRP group (*p* < 0.05). Compared with the control, there were significant differences in the motor function recovery rating of the common peroneal and tibial nerves at 3 and 6 months after treatment in the PRP group (*p* < 0.05) (Table 2). Compared with the baseline, there were significant differences in the motor conduction velocity of the common peroneal nerve and CSA at 1, 3, and 6 months; motor conduction velocity of the tibial nerve at 3 and 6 months; sensory conduction velocity of the common peroneal and tibial nerves at 6 months after treatment in the PRP group (*p* < 0.05); compared with the control, there were significant differences in the motor conduction velocity of the common peroneal nerve at 3 and 6 months, motor conduction velocity of the tibial nerve, and sensory conduction velocity of the common peroneal nerve at 6 months, and CSA at 1 month after treatment in the PRP group (*p* < 0.05) (Table 3).

## 7. Discussion

Studies have shown that repair after peripheral nerve injury requires two phases. The first is the release of chemokines, cytokines, and growth factors by Schwann cells, which promotes the removal of necrotic tissue and provides an environment for regeneration. The second stage is the dedifferentiation and proliferation of Schwann cells, which are then arranged to form Bungner's bands on the basal layer of fibronectin and laminin to meet the growth cones emanating from the regenerating axon and rebuild the axon [17–21]. In vitro and in vivo studies [20, 22–24] have shown that PRP modulates the inflammatory response after injury, promotes macrophage phagocytosis, antigen presentation, and transformation, and contains a variety of cytokines that promote the proliferation and migration of the vascular endothelium to provide a blood supply for nerve regeneration. PRP contains a high concentration of growth factors, which can promote the proliferation and migration of Schwann cells, thereby promoting tissue repair and regeneration. Thus, in basic research, PRP is an indispensable treatment modality for promoting rapid peripheral nerve repair.

In clinical practice, PRP has been studied for the treatment of peripheral nerve injuries for more than 10 years, but has focused on peripheral neuropathy of the upper extremities. A recent systematic evaluation and network meta-analysis of randomized controlled trials showed that PRP injections were the most recommended treatment compared with 5% dextrose and corticosteroid injections. Meanwhile [25], intraneural and perineural injections of PRP were administered after surgical release of the compressed nerves [26–28], and the results showed that patients' pain was reduced, function improved, and long-term efficacy was significant. Tabrizi et al. [29] conducted a randomized double-blind controlled trial and showed that PRP is effective in treating injured alveolar nerves. Sánchez [30] successfully treated a patient with post-traumatic peroneal nerve palsy using PRP under ultrasound guidance. Kuffler et al. [31] successfully treated a patient with a 12-cm-long ulnar nerve defect who had been injured for 3.25 years with a fibrin nerve catheter filled with PRP.

According to existing research on peripheral nerve injury [32, 33], the disease is predominantly male, and the clinical symptoms are mainly motor and sensory dysfunction, including subjective functional scales and objective neurophysiological, ultrasound, and radiological examinations. Patients with complete injuries are treated with surgery, and patients with incomplete injuries can be treated conservatively, including rehabilitation, medication, and biotherapy. PRP is used as a biological treatment. To validate the safety and efficacy of the clinical use of PRP, we designed this randomized controlled clinical trial to recruit 30 male patients with unilateral sciatic nerve incomplete injury and divided them into two groups. The control group received 12 weeks of rehabilitation training, and the PRP group received ultrasound-guided PRP injections combined with rehabilitation training, with one injection per week and five injections, with a follow-up of 6 months, and the safety and efficacy of PRP were comprehensively assessed by using neuroelectrophysiology, ultrasound, and the Motor and Sensory Functional Recovery Rating Scale of BMRC. The results showed that 30 patients had no shedding and no serious adverse reactions, and successfully completed this clinical study and follow-up, which ensured that the quantity and quality of the study data met the statistical requirements. Statistical analysis revealed that there were statistically significant differences in motor conduction velocity of the common peroneal nerve and CSA at 1 month (*p* < 0.05), and there were no significant differences in MFRRT and SFRRT, motor and sensory conduction velocity of the tibial nerve, or sensory conduction velocity of the common peroneal nerve at 1 month after treatment in PRP (*p* > 0.05). There were significant differences in the MFRRT of the common peroneal nerve, motor conduction velocity of the tibial nerve at 3 and 6 months, sensory conduction velocity of the common peroneal and tibial nerves, and MFRRT and SFRRT of the tibial nerve at 6 months after treatment (*p* < 0.05). The results showed that in the early repair of incomplete sciatic nerve injuries, PRP treatment did not improve motor and sensory dysfunction in patients; however, neurophysiological and ultrasound imaging examinations showed partial improvement, implying that PRP may be partially effective in the early repair of incomplete sciatic nerve injuries. During follow-up, the motor and sensory function of the sciatic nerve of the patients treated with PRP showed some improvement, which was consistent with the neurophysiological and ultrasonographic data, implying that PRP was effective in treating incomplete sciatic nerve injuries and the efficacy could be maintained.

## 8. Limitations

This study was constrained by multiple factors, such as funding, the number of patients in our department, time, and ethical requirements for clinical research. The study subjects were not grouped according to the degree of nerve damage. The study was not designed for a multicenter, large-sample, triple-blind, single-factor intervention, and long-term follow-up. It did not observe the changes in PRP-related inflammatory mediators, cytokines, and growth factors in the blood of patients after treatment. It did not carry out correlation analyses of objective and subjective outcome indices. Therefore, it was not possible to observe the benefits of PRP treatment in patients with different degrees of nerve injury, to prove the clinical efficacy of PRP-only treatment, or to explore the possible mechanism of PRP in the early repair and long-term maintenance of SNI.

In the future, we will conduct a multicenter, large-sample, triple-blind randomized controlled trial. Eligible patients with mild to moderate peripheral nerve injury will be enrolled and randomized using computer-generated block randomization with stratification. The control group will receive rehabilitation training, while the treatment group will undergo PRP injection. A long-term follow-up of 12 months or more will be implemented to evaluate primary outcome measures, including clinical response rate, pain relief, and functional recovery. Additionally, blood samples will be collected before and after treatment to analyze PRP-related cytokines and growth factors, aiming to investigate the potential mechanisms of action of PRP in treating peripheral nerve injury.

## 9. Conclusion

PRP may be partially effective in the early repair of incomplete sciatic nerve injuries, and its efficacy can be maintained.

## Figures and Tables

**Table 1:** Baseline demographic and clinical characteristics of study patients.

Evaluation metric	PRP group (*n* = 15)				Control group (*n* = 15)				*p*-Value^a^
Age (years)	28.8 ± 1.83				29.6 ± 1.84				0.76
Body height (cm)	176.26 ± 1.43				175.8 ± 1.39				0.81
Body weight (kg)	76.06 ± 1.48				76 ± 1.49				0.97
Duration (day)	43.73 ± 4.3				45.86 ± 4.63				0.73
Treatment site	—				—				0.45
Left (*n*) (%)	9 (60%)				7 (46.67%)				—
Right (*n*) (%)	6 (40%)				8 (53.33%)				—

	**Excellent**	**Good**	**Moderate**	**Poor**	**Excellent**	**Good**	**Moderate**	**Poor**	

MFRRT (CPN)	4	5	4	2	4	4	3	4	0.98
MFRRT (TN)	4	5	2	4	4	6	1	4	>0.99
SFRRT (CPN)	4	6	2	3	4	6	2	3	>0.99
SFRRT (TN)	4	5	3	3	4	6	2	3	>0.99

MCV(CPN) (m/s)	20.73 ± 4.65				19.13 ± 4.85				0.96
MCV (TN) (m/s)	22.53 ± 5.08				24.73 ± 4.84				0.95
SCV (CPN) (m/s)	25.2 ± 4.89				24.86 ± 4.81				0.96
SCV (TN) (m/s)	23.0 ± 5.14				24.4 ± 4.78				0.97
CSA (mm^2^)	0.46 ± 0.01				0.46 ± 0.01				>0.99

^a^Paired *t*-test, chi-square test, or Fisher's exact test.

**Table 2:** Comparison of changes in motor and sensory function recovery rating table of BMRC between both groups.

Evaluation metric	PRP (*n* = 15)				*p*-Value^a^	Control (*n* = 15)				*p*-Value^a^	*p*-Value^b^
	Excellent	Good	Moderate	Poor		Excellent	Good	Moderate	Poor		
MFRRT (CPN)	4	5	4	2	—	4	4	3	4	—	—
1-month	5	6	4	—	0.3	3	7	2	3	0.73	0.23
3-month	7	5	3	—	<0.05^a^	3	7	3	2	0.27	<0.05^b^
6-month	9	6	—	—	<0.05^a^	4	6	3	2	0.46	<0.05^b^
MF (TN)	4	5	2	4	—	4	6	1	4	—	—
1-month	3	7	2	3	0.8	4	6	2	3	0.9	0.86
3-month	6	5	2	2	0.3	3	7	3	2	0.86	<0.05^b^
6-month	6	7	2	0	<0.05^a^	3	7	3	2	0.86	<0.05^b^
SFRRT (CPN)	4	6	2	3	—	4	6	2	3	—	—
1-month	4	6	3	2	0.85	3	7	2	3	0.86	0.71
3-month	4	7	2	2	0.7	3	7	3	2	0.86	0.69
6-month	6	6	3	0	0.17	6	5	2	2	0.47	0.54
SFRRT (TN)	4	5	3	3	—	4	6	2	3	—	—
1-month	4	6	2	3	0.85	4	7	2	2	0.86	0.96
3-month	4	7	2	2	0.59	4	7	2	2	0.86	0.57
6-month	7	5	3	—	<0.05^a^	5	6	2	2	0.59	0.45

^a^Two-way ANOVA multiple comparisons for intragroup data (mean, intragroup).

^b^Paired *t*-test (mean difference, intergroup).

**Table 3:** Comparison of changes in CSA and electrophysiological study between both groups.

	PRP group (*n* = 15)	*p*-Value^a^	Control group (*n* = 15)	*p*-Value^a^	*p*-Value^b^
	Mean ± SE		Mean ± SE		
MCV (CPN) (m/s)	20.73 ± 4.65	—	19.13 ± 4.85	—	
1-month	28.26 ± 4.72^a^	<0.05^a^	24 ± 4.63	0.32	0.31
3-month	31.53 ± 4.42	<0.05^a^	24.66 ± 4.73	0.24	<0.05^b^
6-month	41.26 ± 2.15^a^	<0.05^a^	26.26 ± 5.11	<0.05^a^	<0.05^b^
MCV (TN) (m/s)	22.53 ± 5.08	—	24.73 ± 4.84	—	
1-month	25.8 ± 4.97	0.43	24.66 ± 4.77	0.94	0.89
3-month	29.2 ± 4.78	<0.05^a^	25 ± 4.84	0.91	0.35
6-month	33.2 ± 1.31	<0.05^a^	25.26 ± 4.92	0.83	<0.05^b^
SCV (CPN)(m/s)	25.2 ± 4.89	—	24.86 ± 4.81	—	
1-month	26.06 ± 5.01	0.84	24.2 ± 4.83	0.91	0.76
3-month	27.46 ± 4.56	0.41	24.46 ± 4.72	0.89	0.46
6-month	32.8 ± 4.6	<0.05^a^	29.33 ± 4.92	0.12	<0.05^b^
SCV (TN)(m/s)	23.0 ± 5.14	—	24.4 ± 4.78	—	
1-month	26.93 ± 5.19	0.35	24.53 ± 4.73	0.95	0.46
3-month	28.73 ± 4.79	0.14	26.2 ± 5.04	0.56	0.48
6-month	33.8 ± 4.74	<0.05^a^	29.86 ± 5.05	0.13	0.23
CSA (cm^2^)	0.46 ± 0.01	—	0.46 ± 0.01	—	
1-month	0.55 ± 0.02^ab^	<0.05^a^	0.48 ± 0.01	0.87	<0.05^b^
3-month	0.54 ± 0.02^a^	<0.05^a^	0.51 ± 0.01	0.54	0.55
6-month	0.54 ± 0.02^a^	<0.05^a^	0.51 ± 0.01	0.54	0.55

^a^Two-way ANOVA multiple comparisons for intragroup data (mean, intragroup).

^b^Paired t-test (mean difference, intergroup).

## Data Availability

All data generated and analyzed in this study have been organized and will be deposited in the Figshare repository within 1 month after the official publication of this article. Once deposited, the data will be publicly accessible via the assigned DOI, which will be updated in the published version of the manuscript. For urgent inquiries about data access prior to deposition, please contact the corresponding author at wuweizj@163.com.
